# Supplementary material for: Ratiometric sensing of BiP-client versus BiP levels by the unfolded protein response determines its signaling amplitude
Source: eLife. 2017 Dec 18;6:e27518. doi: 10.7554/eLife.27518 (PMC5792092; doi:10.7554/eLife.27518)
Supplement: Transparent reporting form [file elife-27518-transrepform.pdf]

1st Floor
24 Hills Road
Cambridge CB2 1JP, UK

P 01223 855340
W elifesciences.org
T @elife

eLife's transparent reporting form

We encourage authors to provide detailed information *within their submission* to facilitate the interpretation and replication of experiments. If you have any questions, please contact us: editorial@elifesciences.org.

Sample-size estimation

- You should state whether an appropriate sample size was computed when the study was being designed
- You should state the statistical method of sample size computation and any required assumptions
- If no explicit power analysis was used, you should describe how you decided what sample (replicate) size (number) to use

Please outline where this information can be found within the submission (e.g., page numbers or figure legends), or explain why this information doesn't apply to your submission:

All assays were repeated as independent biological replicates where appropriate. The number of replicate experiments were chosen such to be appropriate for robustly defining to what extent differences between conditions were reproducible, as detailed in the figure legends.
Technical replicates were used for proteomics (Fig 6) and for pulse-chase experiments (Fig 9C), since for these time-course based experiments biological replicates were less informative as the pace of the kinetics slightly differed between experiments even though the kinetics were then identical (but shifted with time).

Replicates

- You should report how often each experiment was performed
- You should include a definition of biological versus technical replication
- The data obtained should be provided and sufficient information should be provided to indicate the number of independent biological and/or technical replicates
- If you encountered any outliers, you should describe how these were handled
- Criteria for exclusion/inclusion of data should be clearly stated
- High-throughput sequence data should be uploaded before submission, with a private link for reviewers provided (these are available from both GEO and ArrayExpress)

Please outline where this information can be found within the submission (e.g., page numbers or figure legends), or explain why this information doesn't apply to your submission:

eLife Sciences Publications, Ltd is a limited liability non-profit non-stock corporation incorporated in the State of Delaware, USA, with company number 5030732, and is registered in the UK with company number FC030576 and branch number BR015634 at the address 1st Floor, 24 Hills Road, Cambridge CB2 1JP | August 2014

The number of biological or technical replicates are given in each of the figure legends or for the proteomics data in the legend of Supplementary File 1

Statistical reporting

- Statistical analysis methods should be described and justified
- Raw data should be presented in figures whenever informative to do so (typically when N per group is less than 10)
- For each experiment, you should identify the statistical tests used, exact values of N, definitions of center, methods of multiple test correction, and dispersion and precision measures (e.g., mean, median, SD, SEM, confidence intervals; and, for the major substantive results, a measure of effect size (e.g., Pearson's r, Cohen's d)
- Report exact p-values wherever possible alongside the summary statistics and 95% confidence intervals. These should be reported for all key questions and not only when the p-value is less than 0.05.

Please outline where this information can be found within the submission (e.g., page numbers or figure legends), or explain why this information doesn't apply to your submission:

Statistical analyses as well as sample sizes and tests performed are stated in the figure legends and/or in source data files.

(For large datasets, or papers with a very large number of statistical tests, you may upload a single table file with tests, Ns, etc., with reference to page numbers in the manuscript.)

Additional data files ("source data")

- We encourage you to upload relevant additional data files, such as numerical data that are represented as a graph in a figure, or as a summary table
- Where provided, these should be in the most useful format, and they can be uploaded as "Source data" files linked to a main figure or table
- Include model definition files including the full list of parameters used
- Include code used for data analysis (e.g., R, MatLab)
- Avoid stating that data files are "available upon request"

Please indicate the figures or tables for which source data files have been provided:

We provide source data for the following Figures:<br>1C; 2F; 2J; 3D; 4B; 4D; 5; 6; 7B; 7C; 8B; 8D; 8E; 8F; 9B; 9C; 9D; 9E; 10B; 10D;10E; 11B; 11D; 11E; 11I; as well as for Figure 9 supplement 1
